# Implementation research on common cancers (lung, breast, and colorectal) in Asia – a systematic review

**DOI:** 10.3389/fonc.2025.1671298

**Published:** 2025-11-07

**Authors:** Ansuman Panigrahi, Swati Sambita Mohanty, Purnashashi Behera, Rutuparna Sibani Dandsena, Priyanka Sahu, Sanghamitra Pati

**Affiliations:** Indian Council of Medical Research (ICMR)-Regional Medical Research Centre, Bhubaneswar, Odisha, India

**Keywords:** lung cancer, breast cancer, colorectal cancer, implementation research, Asia, systematic review

## Abstract

**Introduction:**

Implementation research is crucial for implementing evidence-based interventions in real-world settings. This study aims to assess and collate existing evidence on implementation research related to common cancers (lung, breast, and colorectal) conducted in Asia.

**Methods:**

A comprehensive literature search was conducted using databases, including PubMed, Embase, ScienceDirect, EBSCO, Web of Science, and Scopus, covering publications from 2004 to 2024. Additionally, free search engines and repositories, such as Google Scholar and Shodhganga, were searched to identify other relevant unpublished studies. A systematic review, adhering to the PRISMA 2020 guidelines, was conducted. From 5750 articles, 11 studies were included that specifically investigated implementation strategies for cancer interventions in Asian populations.

**Findings:**

The review included eleven studies; four implementation studies on lung and breast cancers, and three on colorectal cancers. The included studies explored various interventions to improve cancer care, including training, awareness, and access as key implementation barriers. Context-specific strategies were crucial for successful adoption and sustainability. Most studies evaluated reach, acceptability, feasibility, adoption, fidelity, implementation cost, appropriateness, and sustainability, offering valuable insights into implementation research.

**Interpretation:**

Implementation research on common cancers (lung, breast, colorectal) in Asia is very limited, underscoring the necessity of tailored implementation strategies to integrate cancer care interventions in Asia effectively.

**Systematic Review Registration:**

https://www.crd.york.ac.uk/prospero/, identifier PROSPERO CRD42024542247.

## Highlights

- Implementation research focused on common cancers in Asia
- Emphasizes the need for context-specific strategies
- Employs a robust search strategy
- Follows PRISMA 2020 guidelines
- Covers relevant studies published between 2004 and 2024

## 1 Introduction

Cancer continues to be a major global health concern, with many types still lacking a definitive cure (1) Click or tap here to enter text. Despite significant progress in diagnosis and treatment, current strategies to reduce cancer-related mortality remain inadequate. Stronger public health initiatives are therefore needed, especially those that address not only the biomedical but also the psychosocial and mental well-being aspects of patients (2, 3). The burden of cancer is multifaceted, and prevention remains pivotal. Each year, millions of lives are lost and health systems are strained, while the translation of research advances into tangible patient benefit continues to pose significant challenges (4).

With a population of 4.3 billion, set to grow by another billion by 2050, Asia is experiencing rapid aging. The proportion of people aged 65 and older is projected to double by 2030, increasing cancer risk (4). Together with lifestyle changes, urbanization, dietary shifts, increasing obesity, tobacco and alcohol use, and chronic infections, population aging is fuelling a rising cancer burden across the diverse Asian continent, posing a significant public health challenge (5, 6). Cancer is the second leading cause of death in the Asia Pacific, after cardiovascular diseases, accounting for millions of deaths annually. India alone reports over a million new cases each year (7).

Among all cancers, lung, breast, and colorectal cancers are the most prevalent across Asia (7). Men predominantly suffer from lung cancer, while breast cancer is more prevalent among women (7). Lung cancer remains the leading cause of cancer-related deaths, followed by breast and colorectal cancers (8). The surge in colorectal cancer (CRC) incidence, particularly in transitioning economies, is largely driven by westernized diets and sedentary lifestyles (9–11). Targeted prevention and early detection are vital, necessitating implementation research (IR) (11).

Implementation research aims to bridge the gap between cancer research and practice by integrating proven interventions into real-world healthcare (12). IR identifies effective, replicable strategies, considering context and intervention characteristics, to maximize cancer control impact (13). The increasing incidence of cancer in Asia underscores a pressing need for a comprehensive understanding of evidence-based intervention implementation in real-world settings. Given the vast heterogeneity in healthcare systems, economic capacities, and cultural practices across Asia, the implementation of cancer interventions cannot be generalized. Variation in healthcare systems, economic resources, and cultural norms across Asia requires context-specific implementation of cancer interventions. In low-resource settings, task shifting and decentralized screening are appropriate; where infrastructure is strong, specialist-led pathways are feasible. Financing should use public reimbursement and price negotiation in universal-coverage systems, and targeted subsidies where out-of-pocket spending is high. Outreach must be linguistically and culturally adapted to improve uptake. These context-specific choices shape policy on eligibility, coverage, procurement and regulation, workforce and infrastructure investment. Aligning implementation strategies with these policies is necessary to translate efficacy into real-world, equitable, and sustainable cancer control across Asia.

This systematic review aims to characterize existing implementation research on lung, breast, and colorectal cancers in Asia and to assess its potential for effective cancer prevention and control. We hypothesize that implementation research addressing psychosocial and behavioral components can enhance cancer care delivery in the region. The objective of this systematic review was to identify evidence-based implementation approaches by assessing and collating existing implementation research related to lung, breast, and colorectal cancers conducted in Asia.

## 2 Methods

The systematic review protocol was designed utilizing the Preferred Reporting Items for Systematic Reviews and Meta-Analyses (PRISMA-P 2015) guidelines and registered with PROSPERO (Registration No: CRD42024542247) (14). The study selection, data screening, analysis, and reporting processes were conducted following PRISMA 2020 guidelines, reflecting current best practices in systematic review methodology. The entire review process was guided by the PICO framework, ensuring a focused and structured approach to addressing the research question.

### 2.1 Inclusion and exclusion criteria

The specific search strategy will be adapted for each database. The detailed eligibility criteria in this systematic review are given in **Table 1**. The scope of this study includes peer-reviewed English-language articles with quantitative, qualitative, or mixed-method study designs that were published between January 2004 and July 2024. These studies were conducted in Asian populations and focused on the implementation of interventions for common cancers (lung, breast, and colorectal). The PICO (Population, Intervention, Comparator, Outcome) framework, which is described below, served as the framework for the systematic review.

**Table 1:** Inclusion and exclusion criteria.

Category	Inclusion criteria	Exclusion criteria
Population	All age groups targeted or participated in implementation research on common cancers (lung, breast, and colorectal) conducted in Asia	Subjects other than human participants
Intervention	Any evidence-based intervention with embedded implementation research conducted in Asia, that focuses on lung, breast, and colorectal cancers	Interventions that are not specified in the inclusion criteria
Comparator/Control	As appropriate, we will employ a comprehensive search strategy encompassing relevant comparative studies, including both randomized controlled trials (RCTs) and non-randomized designs	Comparator/control other than those specified in the inclusion criteria
Outcome	Implementation outcomes encompass several key dimensions: acceptability, adoption, appropriateness, feasibility, fidelity, penetration, sustainability, and implementation costs. These outcomes also include reach, implementation, and maintenance, which are crucial for understanding the overall effectiveness and impact of the implementation process	Outcomes beyond those specified in the inclusion criteria were not considered for this study
Study design	Quantitative, qualitative, or mixed methods designs were employed in this review. Quantitative study designs were categorized as experimental and observational. Experimental designs included randomized controlled trials (RCTs) and non-randomized controlled trials (non-RCTs). Observational designs encompassed quasi-experimental designs, Pre-experimental designs such as pre-post, and post-only designs. Additionally, observational designs like cohort studies, cross-sectional studies, and case-control studies were considered.	Non-empirical or primary research included in this study encompassed a variety of sources such as reviews, systematic reviews, meta-analyses, editorials, commentaries, letters to editors, opinion papers, newspapers, study protocols, pilot studies, case reports, and surveys.
Geographic scope	Asia	Areas other than Asia
Time frame	2004-2024	Studies published before 2004

### 2.2 Search strategy (electronic databases)

A preliminary search was conducted to gain an understanding of the existing literature on the topic. This initial exploration helped us develop a robust and comprehensive search strategy. A comprehensive literature search was conducted following the Preferred Reporting Items for Systematic Reviews and Meta-Analyses Literature Search Extension (PRISMA-S) criteria on implementation research for managing the three most prevalent cancers (lung, breast, and colorectal) in Asia (15). Searches were performed in electronic databases such as MEDLINE via PubMed, Embase, CINAHL (Ebsco version), Scopus, ScienceDirect, ProQuest, and Web of Science. Additionally, grey literature was explored through Google Scholar and Shodhganga, related to implementation research. The titles, abstracts, and index terms (keywords) of promising articles were examined to identify additional relevant terms and synonyms. The detailed search strategy, where the search process was refined by incorporating controlled vocabularies like MeSH terms and keywords, expanded the scope of relevant articles identified that were related to:

Implementation research: implementation science, evidence-based practice, translational research, quality improvement, implementation process evaluation, barriers, facilitators

Asia: Asian continent, specific country names (e.g., China, India)

Common cancers: lung cancer, breast cancer, colorectal cancer

**Figure 1:**
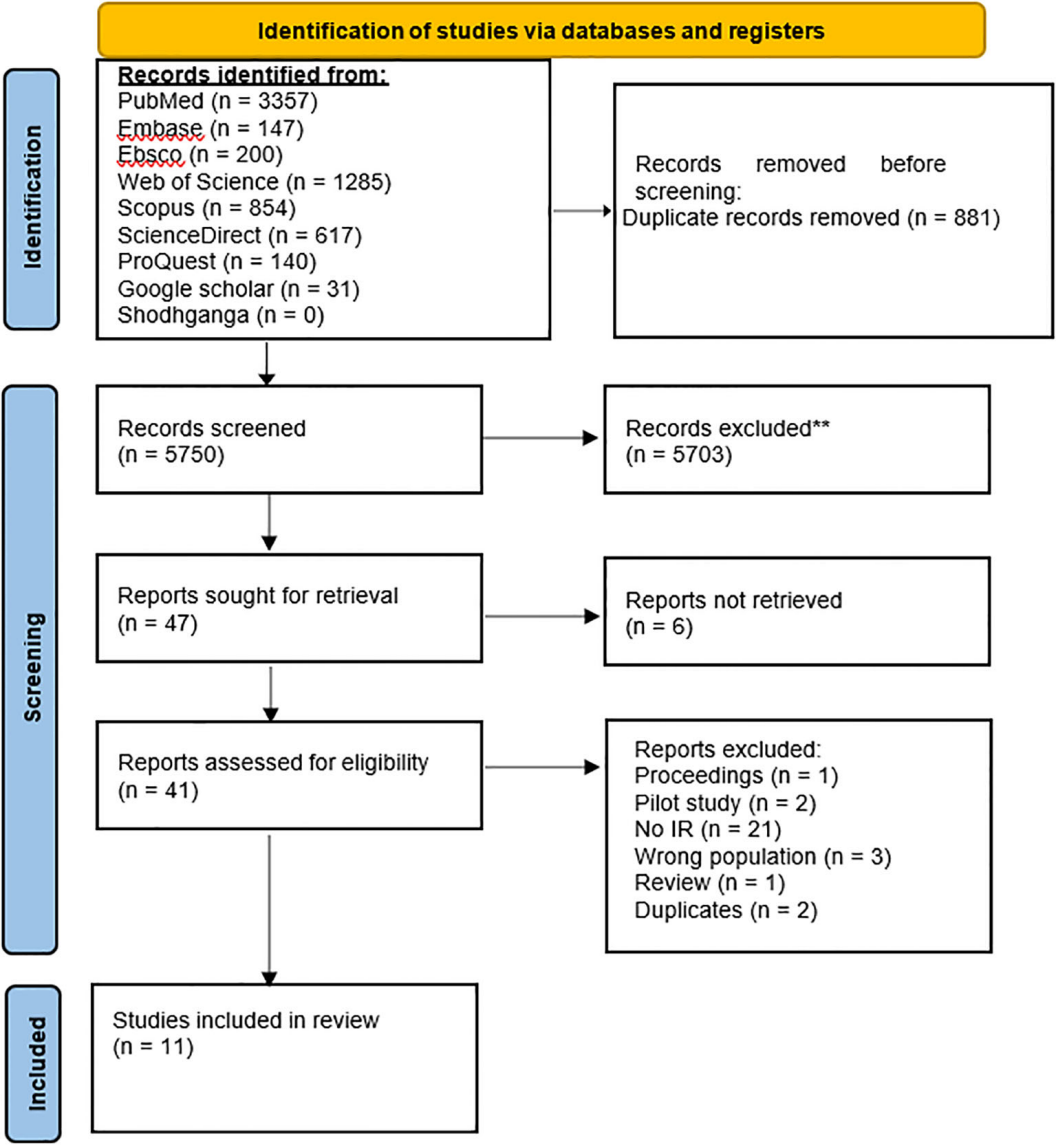
Flow chart for the systematic review procedure. ** Records excluded (n = 5703) were those unrelated to implementation research, and non-human studies.

The Cochrane Database of Systematic Reviews and PROSPERO were extensively searched to find any ongoing systematic reviews on the topic. The PRISMA flow chart outlines the detailed procedure of screening and selecting articles for inclusion in this review, ensuring transparency and replicability (**Figure 1**). The detailed full search syntax for each database is provided in the supplementary **Supplementary Table S1** to ensure transparency and reproducibility.

### 2.3 Study selection and screening process

First, we gathered the studies and converted them into a CSV or RIS format compatible with our software. These were imported into the Rayyan QCRI software to eliminate duplicates (16). Three independent reviewers conducted a two-stage screening process using Rayyan software. In the first stage, titles and abstracts were assessed based on pre-defined inclusion and exclusion criteria. Studies deemed relevant after resolving conflicts were retrieved in full text for further analysis. Following this, full texts were retrieved for all studies meeting the pre-defined criteria. Reasons for exclusion were documented. Any disagreements were settled by consensus or discussion with a designated adjudicator.

### 2.4 Data extraction

Data relevant to implementation research aspects were extracted from the included articles and recorded in a pre-designed Excel spreadsheet for data extraction. This included study characteristics (authors, publication year, study design, etc.), cancer characteristics, the specific intervention implemented, employed implementation strategies, study setting (e.g., primary care, hospital), reported implementation outcomes, and identified facilitators and barriers to implementation.

### 2.5 Risk of bias assessment

The risk of bias assessment in the included studies was conducted to ensure the validity of our findings. The Standards for Reporting Implementation Studies (StaRI) checklist (17) was employed to systematically examine each study for potential biases commonly found in implementation research. Each reported item in a study was scored as “1” while an unreported item was scored as “0”.

### 2.6 Data synthesis

The Synthesis without Meta-analysis (SWiM) guideline was used to guide the synthesis process, focusing on analyzing strategies, contexts, key concepts, methods employed, and reported outcomes (18). Due to the heterogeneity of interventions, diverse outcome measures, contextual factors, and a lack of standardized outcome measures, a meta-analysis was not feasible for this review.

## 3 Results

IR was defined as the studies examining the effective integration of evidence-based interventions in real-world settings. IR included studies on factors influencing implementation, strategies to promote adoption and sustainability, and the processes by which these strategies work. The review considered outcomes such as the acceptability, feasibility, and effectiveness of implementation efforts, as well as their impact on service delivery and patient outcomes, including satisfaction, health status, and symptom management.

The systematic review of IR on common cancers in Asia included four IR studies focusing on lung cancers, four studies exploring IR on breast cancers, and three studies related to colorectal cancers. Most studies addressed the key attributes of IR, including reach, appropriateness, acceptability, feasibility, adoption, implementation cost, sustainability, and fidelity.

A comprehensive search strategy for implementation research on common cancers (lung, breast, colorectal) in Asia yielded a total of 5750 articles. After a thorough evaluation, 41 articles were selected for in-depth review. Ultimately, 11 articles that met the inclusion criteria were incorporated into the review. The key characteristics of the included studies (n=11) regarding lung, breast, and colorectal cancer are presented in **Tables 2**, **3**, **4**. Most research was conducted and published between 2009 and 2023. The study designs were diverse, including six (54.5%) studies with cohort, quasi-experimental with pre- and post-intervention, RCT, single-arm pre- and post, and mixed-methods. Whereas, five (45.5%) studies did not specify study designs. Out of 11 included studies, six (54.5%) were conducted in China, three (27.3%) in Malaysia, one (9.1%) in Taiwan, and 1 (9.1%) in India.

**Table 2:** Characteristics of the included studies regarding lung cancer (n = 4).

Author	Country	Type of cancer	Study design	Purpose	Intervention	Implementation strategy	Implementation outcome	Results
Zhang (2019) (19)	China	Lung	–	To promote evidence-based practice in assessing and managing lung cancer-associated cough within the Department of Radiotherapy of Nanfang Hospital of Southern Medical University	JBI Practical Application of Clinical Evidence System (JBI PACES) and Getting Research into Practice (GRiP) program	Education and training, Quality assurance	Reach Adoption Sustainability	High implementation: 93% of strategies were implemented successfully. Treatment: 90% of patients received treatment for reversible cough causes. Symptomatic therapy: 80% received stepwise symptomatic therapy. Cough suppression: 70% of patients/caregivers received training.
Yang (2017) (20)	Taiwan	Lung	Cohort	To estimate quality-adjusted life expectancy (QALE), loss of QALE, and lifetime healthcare expenditures for lung cancer patients in Taiwan	Low-dose computed tomography screening	Adjusting EYLL through lead-time bias, pathology, and stage-specific proportions for CT screening and radiography	Implementation cost	Incremental costs: US $22,755 per person. Cost-effectiveness ratio: US $19,683 per QALY (based on loss-of-QALE). Potential cost reduction: The ratio could decrease to US $10,947 per QALY with a stage distribution similar to the NELSON trial.
Xiao (2023) (30)	China	Lung	Mixed-method RCT	To evaluate the fidelity of intervention delivery and identify factors influencing its success and the impact of FDT	Family-oriented dignity therapy (FDT)	Randomized allocation of patients, Checklist for intervention delivery data, and qualitative interviews	Fidelity	High fidelity: Intervention implemented with minor deviations from the protocol. Socioeconomic factors: Higher education and income correlated with lower existential distress (H = 12.20, P = 0.030) and higher spiritual well-being (H = 16.310, P = 0.031)
Ghoshal (2021) (21)	India	Lung	–	To assess the rate of early palliative care referrals for all new outpatients with metastatic lung cancer in a premier cancer center in India	Early Palliative Care Referral Program	Education, information such as Process mapping, root cause analysis, Quality improvement interventions, Care coordination	Reach Adoption Sustainability Feasibility Appropriateness	EPC referral increase: Early palliative care (EPC) referrals increased from 50% to 75% post-intervention. Sustained improvement: Referral rates remained consistently higher over time. Effective intervention bundle: A simple intervention bundle successfully increased palliative care utilization without requiring additional resources. Statistical significance: The increase in referrals was statistically significant (mean difference = 12.64, standard deviation = 10.13, 95% confidence interval = 22.01–3.29, P = 0.016).

**Table 3:** Characteristics of the included studies regarding breast cancer (n = 4).

Author	Country	Type of cancer	Study design	Purpose	Intervention	Implementation strategy	Implementation outcome	Results
Ma (2012) (23)	China	Breast	quasi-experimental design with pre- and post-intervention	To examine the impact of a workplace intervention on increasing breast cancer screening rates	Workplace-based educational program guided by the Health Belief Model (HBM) and Social Cognitive Theory (SCT)	Education based on HBM constructs, recruitment facilitated by workplace union leaders, access to mammograms, and financial support	Reach Adoption Appropriateness	Increased Mammography Uptake: Intervention shows a significant increase in mammography use from 10.3% to 72.6% at 6-month follow-up (P < 0.001). High Uptake Among Non-Screened Participants: Nearly 73% of participants without prior mammograms received one post-intervention. Improved Health Beliefs: Significant improvements were observed in 8 out of 10 items related to perceived susceptibility, disease severity, benefits, barriers, and self-efficacy.
Yeoh (2018) (31)	Malaysia	Breast	–	To evaluate the feasibility of patient navigation in a state-run LMIC hospital and assess its impact on diagnostic and treatment timeliness within the first year of implementation.	Patient Navigation (PN) program	Employing multilingual, trained hospital nurses as navigators	Fidelity Appropriateness	Improved diagnostic timeliness: Significant improvements in the speed of diagnosis. Reduced treatment defaults: Lower rates of patients discontinuing treatment. Enhanced communication: Improved communication of news to breast cancer patients.
Schliemann (2020) (32)	Malaysia	Breast	Quasi-experimental study	To assess the impact of a mass media campaign on increasing breast cancer symptom awareness and screening uptake	A culturally adapted mass media campaign (TV, radio, print media, and social media). Be Cancer Alert Campaign (BCAC)	Study population and sampling methods, patient and public involvement, data collection and questionnaire development, BCAC-BC mass media campaign, social media monitoring	Fidelity Reach Appropriateness	The BCAC-BC was implemented as planned with minor deviations (TV advertisement shortened). Knowledge improvement: Significant improvements in six BC symptoms (unprompted), and three BC symptoms (prompted). Demographic impact: Women aged >70 years, had lower exposure to campaign materials and mammogram rates (5%).
Schliemann (2023) (25)	Malaysia	Breast	Randomized controlled trial (RCT)	To design, implement and evaluate an intervention to improve CBE screening uptake and BC symptom recognition in Malaysia	mHealth, community education, and navigation	mHealth tools and community health workers	Reach Adoption Appropriateness	Higher CBE uptake: The intervention group had significantly higher CBE uptake (46%) compared to the control group (4%). Positive CBE follow-up: All women with positive CBEs in the intervention group attended follow-up mammograms (11/11). Income impact: Lower attendance among intervention group women with household income ≥ RM 4,850 compared to those with income < RM 4,850.

**Table 4:** Characteristics of the included studies regarding colorectal cancer (n = 3).

Author	Country	Type of cancer	Study design	Purpose	Intervention	Implementation strategy	Implementation outcome	Results
Luo (2021) (29)	China	Colorectal	A single-arm pre–post-feasibility study design	To evaluate the feasibility, acceptability, and effectiveness of an integrated online and in-person intervention for colorectal cancer (CRC) patients and their caregivers to improve positive coping	Online platform, face-to-face sessions	Blended Learning Format: Combined online and face-to-face sessions to cater to diverse learning styles and preferences. Weekly Reminders: Sent reminders to ensure consistent engagement and completion of dyadic sessions. Face-to-Face Sessions: Conducted biweekly face-to-face meetings for reinforcement and additional support	Reach Acceptability Fidelity Feasibility	High Engagement: Strong recruitment (70.6%), retention (83.3%), and session completion (85%). Online Activity: Dyadic Learning Sessions viewed approximately 609 times. Positive Ratings: Participants reported high levels of usefulness, ease of use, and satisfaction. Improved Outcomes: Small-to-medium improvements in self-efficacy and other outcomes for CRC patients and caregivers.
Gong (2018) (33)	China	Colorectal	–	To implement a community-based colorectal cancer screening program in Shanghai, China	Free initial screening, referral for colonoscopy	Community partnerships, mass media advocacy, health information distribution, informed consent	Reach Adoption Appropriateness	Registrants: 828,302 Shanghai residents registered. Screening Completion: 97.7% (809,528) completed initial screening. Colonoscopy: 71,733 of 180,094 screening-positive participants underwent colonoscopy (39.8% compliance). CRC Detection: 1,630 CRC cases diagnosed (201.35/100,000), with 51.6% in stages 0-1. Decreasing Compliance: Colonoscopy compliance decreased with age and education level.
Meng W (2009) (34)	China	Colorectal	–	To evaluate the impact of a barrier-focused intervention on colonoscopy attendance among nonadherent high-risk individuals in a community-based CRC screening program	Multifaceted barriers-focused intervention program	Telephonic interviews, on-site interviews	Reach Fidelity Feasibility	Increased attendance: Colonoscopy attendance increased from 23% to 38% after intervention. Barrier effectiveness: Intervention was more effective for addressing objective barriers than subjective barriers. High completion: Colonoscopy completion rate was high (87.14%) among individuals without barriers.

The included 11 studies on lung, breast, and colorectal cancers employed diverse implementation strategies. These included education programs (19), early detection (20, 21), and supportive/palliative care (21), psychosocial interventions (22), workplace-based education (23), mass media campaigns (24) Click or tap here to enter text., mHealth tools (25), community-based screening (26), barrier-focused interventions (27, 28) and blended online/in-person support (29). These tailored strategies collectively aimed to improve patient care and increase screening rates.

The 11 studies detailed in **Tables 2**-**4** measured various implementation outcomes, with each study focusing on specific outcomes relevant to their interventions and cancer types. In this review, eight (72.7%) studies measured reach, five (45.5%) measured adoption, two (18.2%) measured sustainability, five (45.5%) measured fidelity, three (27.3%) measured feasibility, six (54.5%) measured appropriateness, and only one (9.1%) from Taiwan measured the implementation cost as healthcare expenditures related to lung cancer screening in US dollars. Reach was the most studied implementation outcome, followed by appropriateness, adoption, and fidelity.

### 3.1 Summary of the risk of bias assessment

The STARI checklist, which assesses implementation and intervention details, was used to evaluate the quality of the included studies. The checklist consists of 27 items. The studies generally scored well, implementation received an average score of 15-21, and intervention evaluation scored 15–20 out of 27 StaRI items (**Supplementary Table S2**).

## 4 Discussion

This systematic review represents the first comprehensive examination of implementation research focused on common cancers (lung, breast, colorectal) in Asia revealing critical insights about the translation of evidence-based interventions into real-world cancer care settings. Implementation research is critical for bridging the gap between research findings and real-world practice, elucidating the factors that influence the translation of efficacious interventions into effective clinical practice.

### 4.1 Implementation research on lung cancers in Asia

#### 4.1.1 Symptom management

The study by Zhang et al. demonstrated the effectiveness of evidence-based interventions for improving lung cancer-associated cough management (19). Healthcare organizations should invest in nursing education to ensure that staff are equipped with the knowledge and skills necessary for effective cough management. Patient and caregiver education are essential for empowering individuals to manage their symptoms effectively. A robust quality control system is crucial for maintaining high standards of care and ensuring that best practices are consistently implemented.

#### 4.1.2 Screening/early detection

Yang et al. provided compelling evidence for the cost-effectiveness of low-dose CT screening for lung cancer in high-risk smokers (20). Healthcare systems must prioritize screening programs for high-risk smokers. Clear guidelines and protocols should be established to ensure consistent and accurate screening practices. Additionally, efforts should be made to address participation barriers, such as fear, anxiety, and logistical challenges. By addressing the identified challenges and implementing effective strategies, healthcare systems can improve early detection, enhance treatment outcomes, and ultimately save lives. Low-dose CT screening for high-risk individuals is a cost-effective strategy for early detection of lung cancer.

#### 4.1.3 Psychosocial support

Another study conducted by Xiao et al. provides valuable insights into the implementation and impact of Family-Oriented Dignity Therapy (FDT) for lung cancer patients (30). Addressing socioeconomic disparities and tailoring the intervention to individual patient needs to provide a positive effect on patients’ psychosocial well-being. Providing adequate emotional support, facilitating effective communication between patients and healthcare providers, and offering personalized care maximizes the benefits of FDT that address physical and emotional distress. The quality of life for lung cancer patients and their families can be improved by addressing the identified barriers and promoting the implementation of evidence-based psychosocial interventions. Therefore, FDT can enhance the psychosocial well-being of lung cancer patients and their families.

#### 4.1.4 Palliative care

Similarly, Ghoshal et al. demonstrate the effectiveness of a simple quality improvement project in significantly increasing early palliative care (EPC) referrals for metastatic lung cancer patients (21). Ongoing training, clear roles within the team, and integration of palliative care can facilitate timely and appropriate referrals. The potential for quality improvement initiatives to enhance patient-centered care and patient outcomes can increase EPC referrals and improve the quality of life for patients with advanced cancer.

### 4.2 Implementation research on breast cancer in Asia

#### 4.2.1 Workplace-based interventions

Ma et al. reported the effectiveness of workplace-based interventions in increasing breast cancer screening rates among Chinese women (23). Integrating health promotion programs into workplaces can reach a wider audience and promote positive health behaviors. However, cultural perceptions and logistical challenges can hinder the long-term impact of these interventions. To overcome these barriers, ongoing education, support, and collaboration with local health authorities are crucial. Empowering women to take control of their health and reduce breast cancer risk requires addressing cultural barriers, leveraging workplace dynamics, and providing tailored education and support. However, leveraging workplace settings to promote health education and screening can significantly increase participation rates.

#### 4.2.2 Patient navigation

The feasibility and effectiveness of patient navigation in improving breast cancer care in a low- and middle-income country (LMIC) setting were described by Yeoh et al. in 2018 (31). The study highlights the importance of culturally sensitive approaches in patient navigation programs. By understanding and addressing cultural and socioeconomic barriers, healthcare providers can tailor interventions to meet the specific needs of diverse patient populations. Patient navigation programs have the potential to significantly improve breast cancer care in LMIC settings by addressing barriers, providing support, and promoting adherence to care pathways.

#### 4.2.3 Mass-media campaigns

Similarly, Schliemann et al. assessed the impact of a mass media campaign on breast cancer symptoms awareness and screening uptake among women in Malaysia (32). Addressing cultural barriers and negative perceptions of screening is crucial for improving breast cancer outcomes. By providing accurate information and addressing misconceptions, healthcare providers can empower women to make informed decisions about their health. Mass media campaigns can be a powerful tool for increasing breast cancer awareness and promoting early detection. By addressing the specific needs of diverse populations and incorporating culturally sensitive messaging, these campaigns can contribute to significant improvements in breast cancer outcomes. Thus, culturally tailored mass-media campaigns can increase awareness, improve knowledge, and encourage help-seeking behaviors.

#### 4.2.4 Community engagement

In 2023, the effectiveness of a mHealth intervention to improve breast cancer screening uptake in rural Malaysia was assessed by Schliemann et al. (25). By leveraging community health workers and providing targeted education and support, increased clinical breast examination (CBE) attendance. The study underlines the significance of addressing barriers to screening, like low income, and tailoring interventions to the specific population needs to improve screening access. Combining technology with community-based approaches improved outreach and screening access, leading to better breast cancer outcomes and reduced health disparities in rural areas. Engaging community health workers and providing targeted education and support can improve access to screening services, especially in rural and underserved areas.

### 4.3 Implementation research on colorectal cancers in Asia

#### 4.3.1 CRC couple support

Luo et al. assessed the feasibility and preliminary efficacy of a blended intervention program for CRC patients and their spousal caregivers (29). The study highlights the importance of combining online and face-to-face support to enhance engagement and improve outcomes. It demonstrated the potential to improve self-efficacy and emotional well-being in both patients and caregivers. By integrating online and traditional support methods, healthcare providers can offer more flexible and accessible support to CRC couples, ultimately improving their quality of life and well-being.

#### 4.3.2 CRC screening

In 2018, Gong et al. studied the implementation and initial results of a community-based CRC screening program in Shanghai, China (33). The study noted the importance of large-scale screening programs in the early detection and prevention of CRC. The study identified several barriers to screening, including limited awareness, accessibility issues, and referral system challenges. Addressing these barriers through effective reminders, proactive registration, and improved referral processes can enhance program effectiveness and reduce the burden of CRC in China and other high-risk populations.

#### 4.3.3 Barrier-focused intervention

In another study, Meng W et al. evaluated a barrier-focused intervention to increase colonoscopy attendance among high-risk individuals for CRC screening in China (27). By addressing both subjective and objective barriers, the intervention significantly improved compliance rates, particularly among individuals with specific high-risk factors. Healthcare providers can develop more effective strategies to improve screening rates by identifying and addressing the underlying reasons for non-adherent individuals, thereby reducing the burden of this disease.

### 4.4 Comparative insights: Asia vs West—strengths and challenges

The high implementation success rates observed in Asian settings, often exceeding 65–93%, underscore the potential of contextually adapted and community-engaged approaches, even where resources are limited. These results compare favorably to Western countries, where although implementation science frameworks (e.g., RE-AIM, CFIR) are well-established, variable real-world performance persists. For example, Zhang et al. (19) in China attained a 93% intervention uptake through robust education strategies, whereas US and European programs, despite higher resources, often struggle with reach due to system complexity and rigid standardization (34, 35). Several factors may drive these differences: more centralized systems with clearer pathways, stronger community interfaces via community health workers and local leaders, greater implementation flexibility, and incentives aligned to uptake. But cross-setting comparisons face biases like different uptake definitions, publication of high-performing sites, and short follow-ups. Asian strategies (community mobilization, simplified pathways) are promising, yet should be tested under Western constraints and assessed for durability, equity, and cost-effectiveness. The family-oriented dignity therapy implemented by Xiao et al. (30) in China achieved 95% fidelity by integrating family members into psychosocial support, contrasting sharply with individual-focused psychosocial interventions predominant in Western countries (36, 37). Higher fidelity likely reflects cultural fit with collectivist norms, clear caregiver roles, and shared responsibility that reduces drop-off. Risks include privacy/autonomy concerns, uneven caregiver capacity, and social-desirability bias. Western adaptations should test family-inclusive models with consent safeguards, caregiver training and burnout screening, and outcomes capturing patient- and family-level effects. The community-based mHealth intervention by Schliemann et al. (25) in Malaysia achieved significant screening uptake (46% vs 4% control) by leveraging community health workers and mobile technology, demonstrating how resource limitations can foster creative solutions. Resource constraints in Asian healthcare systems have paradoxically driven innovative implementation approaches that achieve high efficiency and effectiveness. This contrasts with resource-rich Western implementations that rely on specialized professional staff and expensive infrastructure. Western implementation research, particularly in countries like Germany and the United States, benefits from higher resource availability but often faces challenges with sustainability and scalability (34, 37). The German psycho-oncological care system, while comprehensive and professionally staffed, struggles with geographic disparities and cost-effectiveness concerns (37). Asian implementations, constrained by resource limitations, have developed inherently sustainable models that integrate cancer care into existing community structures and health systems.

Asian models combine digital outreach with local worker support, offering lessons in scaling equity. Only one Asian study assessed implementation costs, compared to more frequent economic analysis in contemporary Western work (35, 38). Comprehensive outcome evaluation including sustainability, fidelity, cost-effectiveness, and adoption is crucial for real-world impact and cross-regional learning. Also, most studies assessed reach and feasibility, while fewer examined cost-effectiveness, sustainability, and patient-centeredness, which are key elements essential for assessing real-world impact.

Based on the findings of our study, strengthening current implementation strategies requires integrating local contextual evidence into policy formulation. Capacity building for health professionals, institutionalization of implementation research within cancer control programs, and development of context-specific monitoring frameworks can enhance sustainability. Strengthening cross-country collaboration across Asian regions will further support the translation of evidence into scalable and equitable cancer control practices.

The findings from this review have significant implications for strengthening national cancer control strategies across Asia. Implementation research serves as a critical bridge between scientific evidence and public health policy, providing context-specific insights that can guide the design, adaptation, and scaling of cancer programs. For instance, countries such as India, through its National Programme for Prevention and Control of Cancer, Diabetes, Cardiovascular Diseases, and Stroke (NPCDCS), and China, through the National Cancer Prevention and Control Plan, have emphasized early detection, screening, and palliative care but implementation gaps persist due to variations in health infrastructure, workforce capacity, and regional inequities. Evidence from this review demonstrates that context-sensitive interventions such as patient navigation, mHealth-supported screening, and community-based outreach can effectively address these gaps by improving reach, adoption, and fidelity within real-world healthcare systems.

Moreover, embedding IR frameworks such as RE-AIM and StaRI into national cancer control monitoring systems can enhance policy responsiveness by enabling continuous evaluation of program effectiveness and sustainability. Policymakers can utilize IR findings to identify barriers to intervention uptake, optimize resource allocation, and adapt interventions to cultural and socioeconomic realities. Integrating these insights into existing cancer control roadmaps will not only strengthen program implementation but also ensure that evidence-based interventions reach the most underserved populations. In this way, IR can transform cancer control policies from being top-down and generic to adaptive, data-driven, and contextually grounded across diverse Asian healthcare settings.

While biomedical advances have improved cancer survival, psychosocial challenges including distress, anxiety, stigma, and reduced quality of life remain major determinants of patient outcomes. Implementation research plays a vital role in translating evidence-based psychosocial interventions into real-world practice, ensuring that cancer care is not limited to clinical management but extends to holistic well-being. Several interventions reviewed in this study, such as family-oriented dignity therapy, patient navigation, and community-based education models, directly address these dimensions by fostering emotional resilience, improving communication, and enhancing coping mechanisms for patients and caregivers. Thus, this review also sought to explore how implementation research in Asia operationalized the concept of patient well-being through psychosocial and supportive care interventions. Emerging evidence suggests that Artificial Intelligence (AI) can augment psychosocial support in cancer care through predictive analytics, virtual counseling, and adaptive intervention designs (39, 40). Incorporating AI into implementation strategies may help personalize interventions, optimize resource allocation, and strengthen patient-provider interactions.

## 5 Limitations

The review has several limitations. First, the focus on English-language, peer-reviewed articles might exclude relevant non-English or unpublished research. Second, inconsistencies in study terminology hindered the categorization of studies. Finally, the limited number of studies prevented a quantitative analysis, such as a meta-analysis, from synthesizing the findings. The diverse nature of interventions and outcome measures may limit the comparability of results across studies. The focus on specific Asian populations may restrict the applicability of results to other regions or demographics. Over half of the included studies are from China, which likely skews findings toward China’s health-system structure, financing, urban service delivery, and cancer screening practices, while underrepresenting the resource constraints, sociocultural norms, and policy or regulatory contexts common in South and West Asia. As a result, estimated feasibility, uptake, and cost-effectiveness may be overstated for settings with weaker primary care, higher out-of-pocket spending, or different care-seeking behaviors in those regions. Future research should focus on underrepresented countries to ensure a more balanced regional understanding. Nearly half of the included studies did not specify their research design, which raises questions about methodological transparency and may influence the reliability of reported outcomes. Restricting to English-language studies may have led to exclusion of significant regional evidence published in local Asian languages. Several implementation initiatives might remain unpublished or available only through institutional or local-language reports, suggesting the need for broader inclusion of non-indexed sources in future reviews.

## 6 Conclusion and recommendation

This systematic review highlights the importance of implementing evidence-based interventions in real-world settings to improve cancer care, particularly for common cancers (lung, breast, and colorectal) in Asia. Focusing on China, Malaysia, Taiwan, and India, the review analyses recent studies investigating key strategies such as early detection, symptom management, psychosocial support, and addressing health disparities. The findings reveal that while diverse implementation strategies exist, significant barriers remain. These include inadequate healthcare provider training, insufficient awareness of evidence-based practices, and limited access to cancer screening and treatment services. Country-specific adaptation of cancer control frameworks is needed for example, patient navigation in Malaysia, palliative care integration in India, and psychosocial support in China can guide localized models.

Implementation research serves a dual purpose: improving cancer prevention (via early detection and screening) and enhancing healthcare delivery (through symptom control and support services). Understanding these distinct goals is crucial as they often require different implementation strategies and target distinct groups of people. While the studies assessed various implementation outcomes, with reach being the most frequently measured, there was a lack of comprehensive evaluation of other important aspects, such as cost-effectiveness, long-term sustainability, and patient-centered approaches.

Compared to Western countries, where implementation science frameworks are well-developed, Asian countries need approaches that are context-specific, culturally sensitive, and resource-feasible. To bridge the gap between evidence and practice in Asian healthcare systems, there’s an urgent need for pragmatic trials, participatory models, and real-world evaluations. Key recommendations to enhance cancer patient and family quality of life include prioritizing nursing education, ensuring quality control, implementing patient-centered care, and raising awareness about the importance of IR among key stakeholders, including government officials, healthcare providers, and researchers. This involves highlighting the need for IR in evaluating the acceptability, feasibility, adaptability, and cost-effectiveness of cancer control programs.

Adapting IR strategies to healthcare systems, we can effectively address context-specific barriers, thereby improving the timeliness of cancer prevention and early detection in real-world settings. Fostering ongoing education, community engagement, and collaboration with local health authorities will be essential to enhance cancer care and screening uptake. Implementing multicomponent interventions and addressing socioeconomic factors will further improve access to cancer screenings.

## Data Availability

The original contributions presented in the study are included in the article/**Supplementary Material**. Further inquiries can be directed to the corresponding author.

## Supplementary material

Supplementary Table 1 Search strategy for one database with search terms.

Supplementary Table 2 Risk of bias assessment.
