# Supplementary material for: Implementation research on common cancers (lung, breast, and colorectal) in Asia – a systematic review
Source: Front Oncol. 2025 Nov 7;15:1671298. doi: 10.3389/fonc.2025.1671298 (PMC12634338; doi:10.3389/fonc.2025.1671298)
Supplement: Supplementary Table 1 — Search strategy for one database with search terms. [file Table1.docx]

**Supplementary Table 1:** Search strategy summary-IR on common cancers (lung, breast, colorectal) in Asia

| **Search strategy 1 – Pubmed** | | |
| --- | --- | --- |
| **Search number** | **Query** | **Results** |
| 1 | ("Implementation Science"[Mesh] OR "Health Plan Implementation"[Mesh]) OR ("Implementation Science*"[Title/Abstract] OR "Health Plan Implementation*"[Title/Abstract] OR Implementation[Title/Abstract] OR implementing[Title/Abstract] OR implemented[Title/Abstract] OR operations[Title/Abstract] OR delivery[Title/Abstract] OR deliver[Title/Abstract] OR "translational science"[Title/Abstract] OR "translational research"[Title/Abstract] OR "Translational Medical Research"[Title/Abstract] OR "quality improvement"[Title/Abstract] OR "task shifting"[Title/Abstract] OR policy[Title/Abstract] OR monitoring[Title/Abstract] OR evaluation[Title/Abstract] OR "implementation process evaluation"[Title/Abstract] OR "impact evaluation"[Title/Abstract] OR "effectiveness, implementation"[Title/Abstract] OR "hybrid research"[Title/Abstract] OR "implementation studies"[Title/Abstract] OR "applied research"[Title/Abstract] OR "practice based research"[Title/Abstract] OR "operational research"[Title/Abstract] OR "applied implementation science"[Title/Abstract] OR "outcome evaluation"[Title/Abstract] OR "evidence based practice"[Title/Abstract] OR "policy implementation"[Title/Abstract] OR "stakeholder engagement"[Title/Abstract] OR "knowledge translation"[Title/Abstract] OR "contextual factors"[Title/Abstract] OR "barriers and facilitators"[Title/Abstract] OR "implementation strategies"[Title/Abstract] OR "implementation outcomes"[Title/Abstract] OR "real world implementation"[Title/Abstract] OR "implementation framework"[Title/Abstract] OR "Implementation fidelity"[Title/Abstract] OR "Implementation interventions"[Title/Abstract] OR "Implementation research methods"[Title/Abstract]) | 37,04,045 |
| 2 | ("Breast Neoplasms"[Mesh] OR "Lung Neoplasms"[Mesh] OR "Colorectal Neoplasms"[Mesh]) OR ("Breast Neoplasm*"[Title/Abstract] OR "Neoplasm*, Breast"[Title/Abstract] OR "Breast Tumors*"[Title/Abstract] OR "Breast Cancer*"[Title/Abstract] OR "Cancer*, Breast"[Title/Abstract] OR "Mammary Cancer*"[Title/Abstract] OR "Cancer, Mammary*"[Title/Abstract] OR "Malignant Neoplasm of Breast*"[Title/Abstract] OR "Breast Malignant Neoplasm*"[Title/Abstract] OR "Breast Malignant Tumor*"[Title/Abstract] OR "Cancer of the Breast*"[Title/Abstract] OR "Human Mammary Carcinoma*"[Title/Abstract] OR "Breast Carcinoma"[Title/Abstract] OR "Carcinoma*, Breast"[Title/Abstract] OR "mammary neoplasm"[Title/Abstract] OR "breast malignancy"[Title/Abstract] OR "Pulmonary Neoplasm*"[Title/Abstract] OR "Neoplasms, Lung*"[Title/Abstract] OR "Lung Neoplasm*"[Title/Abstract] OR "Neoplasm*, Lung"[Title/Abstract] OR "Neoplasm, Pulmonary*"[Title/Abstract] OR "Lung Cancer*"[Title/Abstract] OR "Cancer, Lung*"[Title/Abstract] OR "Pulmonary Cancer*"[Title/Abstract] OR "Cancer, Pulmonary*"[Title/Abstract] OR "Cancer of the Lung*"[Title/Abstract] OR "pulmonary carcinoma"[Title/Abstract] OR "lung carcinoma"[Title/Abstract] OR "bronchogenic carcinoma"[Title/Abstract] OR "bronchial carcinoma"[Title/Abstract] OR "bronchial cancer"[Title/Abstract] OR "pulmonary malignancy"[Title/Abstract] OR "lung malignancy"[Title/Abstract] OR "pulmonary tumor"[Title/Abstract] OR "bronchogenic tumor"[Title/Abstract] OR "bronchial tumor"[Title/Abstract] OR "respiratory cancer"[Title/Abstract] OR "pulmonary adenocarcinoma"[Title/Abstract] OR "small cell lung cancer"[Title/Abstract] OR "non-small cell lung cancer"[Title/Abstract] OR "adenocarcinoma of the lung"[Title/Abstract] OR "squamous cell carcinoma of the lung"[Title/Abstract] OR "bronchoalveolar carcinoma"[Title/Abstract] OR mesothelioma[Title/Abstract] OR "Colorectal Neoplasm*"[Title/Abstract] OR "Neoplasm*, Colorectal*"[Title/Abstract] OR "Colorectal Tumor*"[Title/Abstract] OR "Tumor, Colorectal*"[Title/Abstract] OR "Colorectal Cancer*"[Title/Abstract] OR "Cancer*, Colorectal"[Title/Abstract] OR "Colorectal Carcinoma*"[Title/Abstract] OR "Carcinoma*, Colorectal*"[Title/Abstract] OR "bowel cancer"[Title/Abstract] OR "colon cancer"[Title/Abstract] OR "rectal cancer"[Title/Abstract] OR "intestinal cancer"[Title/Abstract] OR "colonic carcinoma"[Title/Abstract] OR "rectal carcinoma"[Title/Abstract] OR "large bowel carcinoma"[Title/Abstract] OR "colorectal adenocarcinoma"[Title/Abstract] OR "intestinal carcinoma"[Title/Abstract] OR "colon adenocarcinoma"[Title/Abstract] OR "rectal adenocarcinoma"[Title/Abstract] OR "colorectal neoplasm"[Title/Abstract] OR "large intestine cancer"[Title/Abstract] OR "lower gastrointestinal cancer"[Title/Abstract] OR "gastrointestinal malignancy"[Title/Abstract] OR "bowel malignancy"[Title/Abstract] OR "intestinal malignancy"[Title/Abstract]) | 1,123,931 |
| 3 | ("Asia"[Mesh]) OR (Asia[Title/Abstract] OR "Northern Asia"[Title/Abstract] OR "North Asia"[Title/Abstract] OR "Far East"[Title/Abstract] OR "Eastern Asia"[Title/Abstract] OR "East Asia"[Title/Abstract] OR "Western Asia"[Title/Abstract] OR "Southeastern Asia"[Title/Abstract] OR "Southeast Asia"[Title/Abstract] OR "Asia, Central"[Title/Abstract] OR "Central Asia"[Title/Abstract] OR "South* Asia"[Title/Abstract] OR "Asia, South"[Title/Abstract] OR "British Indian Ocean Territory"[Title/Abstract] OR Bangladesh[Title/Abstract] OR India[Title/Abstract] OR Bharat[Title/Abstract] OR "Republic of India"[Title/Abstract] OR Myanma*[Title/Abstract] OR Burma[Title/Abstract] OR "Khmer Republic"[Title/Abstract] OR Cambodia[Title/Abstract] OR Kampuchea[Title/Abstract] OR "People's Republic of China"[Title/Abstract] OR "Mainland China"[Title/Abstract] OR Sinkiang[Title/Abstract] OR "Inner Mongolia"[Title/Abstract] OR "Netherlands East Indies"[Title/Abstract] OR "East Indies"[Title/Abstract] OR "West Irian"[Title/Abstract] OR "New Guinea, Indonesian"[Title/Abstract] OR "New Guinea, West"[Title/Abstract] OR "Indonesian New Guinea"[Title/Abstract] OR "Irian Jaya"[Title/Abstract] OR Timor[Title/Abstract] OR Java[Title/Abstract] OR Bali[Title/Abstract] OR Sumatra[Title/Abstract] OR Celebes[Title/Abstract] OR Sulawesi[Title/Abstract] OR "Malay Archipelago"[Title/Abstract] OR Madura[Title/Abstract] OR "Bonin Islands"[Title/Abstract] OR Malaya[Title/Abstract] OR "Federation of Malaya"[Title/Abstract] OR Sabah[Title/Abstract] OR Sarawak[Title/Abstract] OR "Malay Peninsula"[Title/Abstract] OR "Federal Democratic Republic of Nepal"[Title/Abstract] OR "Islamic Republic of Pakistan"[Title/Abstract] OR Philipines[Title/Abstract] OR "Kingdom of Thailand"[Title/Abstract] OR Siam[Title/Abstract] OR Viet Nam[Title/Abstract] OR "Vietnam, Republic of"[Title/Abstract] OR "North Vietnam"[Title/Abstract] | 1,218,644 |
| 4 | #1 AND #2 | 127,742 |
| 5 | #3 AND #4 | 3357 (included) |
| **Search strategy 2 - EBSCO** | | |
| 1 | TX (“Implementation Science*” OR “Health Plan Implementation*” OR Implementation OR implementing OR implemented OR operations OR delivery OR deliver OR “translational science” OR “translational research” OR “Translational Medical Research” OR “quality improvement” OR “task shifting” OR policy OR monitoring OR evaluation OR “implementation process evaluation” OR “impact evaluation” OR “effectiveness, implementation” OR “hybrid research” OR “implementation studies” OR “applied research” OR “practice-based research” OR “operational research” OR “applied implementation science” OR “outcome evaluation” OR “evidence-based practice” OR “policy implementation” OR “stakeholder engagement” OR “knowledge translation” OR “contextual factors” OR “barriers and facilitators” OR “implementation strategies” OR “implementation outcomes” OR “real world implementation” OR “implementation framework” OR “Implementation fidelity” OR “Implementation interventions” OR “Implementation research methods”) | 22,93,290 |
| 2 | TX (“Breast Neoplasm*” OR “Neoplasm*, Breast” OR “Breast Tumors*” OR “Breast Cancer*” OR “Cancer*, Breast” OR “Mammary Cancer*” OR “Cancer, Mammary*” OR “Malignant Neoplasm of Breast*” OR “Breast Malignant Neoplasm*” OR “Breast Malignant Tumor*” OR “Cancer of the Breast*” OR “Human Mammary Carcinoma*” OR “Breast Carcinoma” OR “Carcinoma*, Breast” OR “Pulmonary Neoplasm*” OR “Neoplasms, Lung*” OR “Lung Neoplasm*” OR “Neoplasm*, Lung” OR “Neoplasm, Pulmonary*” OR “Lung Cancer*” OR “Cancer, Lung*” OR “Pulmonary Cancer*” OR “Cancer, Pulmonary*” OR “Cancer of the Lung*” OR “Colorectal Neoplasm*” OR “Neoplasm*, Colorectal*” OR “Colorectal Tumor*” OR “Tumor, Colorectal*” OR “Colorectal Cancer*” OR “Cancer*, Colorectal” OR “Colorectal Carcinoma*” OR “Carcinoma*, Colorectal*”) | 2,06,397 |
| 3 | TX (Asia OR “Northern Asia” OR “North Asia” OR “Far East” OR “Eastern Asia” OR “East Asia” OR “Western Asia” OR “Southeastern Asia” OR “Southeast Asia” OR “Asia, Central” OR “Central Asia” OR “South* Asia” OR “Asia, South” OR “British Indian Ocean Territory” OR Bangladesh OR India OR Bharat OR “Republic of India” OR Myanma* OR Burma OR “Khmer Republic” OR Cambodia OR Kampuchea OR “People's Republic of China” OR “Mainland China” OR Sinkiang OR “Inner Mongolia” OR “Netherlands East Indies” OR “East Indies” OR “West Irian” OR “New Guinea, Indonesian” OR “New Guinea, West” OR “Indonesian New Guinea” OR “Irian Jaya” OR Timor OR Java OR Bali OR Sumatra OR Celebes OR Sulawesi OR “Malay Archipelago” OR Madura OR “Bonin Islands” OR Malaya OR “Federation of Malaya” OR Sabah OR Sarawak OR “Malay Peninsula” OR “Federal Democratic Republic of Nepal” OR “Islamic Republic of Pakistan” OR Philipines OR “Kingdom of Thailand” OR Siam OR Viet Nam OR “Vietnam, Republic of” OR “North Vietnam”) | 3,73,202 |
| 4 | #1 AND #2 AND #3 | 200 (included) |
| **Search strategy 3 - Embase** | | |
| 1 | ('breast tumor'/exp OR 'bilateral breast neoplasm' OR 'bilateral breast tumor' OR 'bilateral breast tumour' OR 'breast gland tumor' OR 'breast gland tumour' OR 'breast mass' OR 'breast neoplasia' OR 'breast neoplasm' OR 'breast neoplasms' OR 'breast neoplasms, male' OR 'breast tumor' OR 'breast tumorigenesis' OR 'breast tumour' OR 'female breast neoplasm' OR 'female breast tumor' OR 'female breast tumour' OR 'male breast neoplasm' OR 'male breast tumor' OR 'male breast tumour' OR 'mamma tumor' OR 'mamma tumour' OR 'mammary gland neoplasia' OR 'mammary gland neoplasm' OR 'mammary gland tumor' OR 'mammary gland tumorigenesis' OR 'mammary gland tumour' OR 'mammary neoplasia' OR 'mammary neoplasm' OR 'mammary neoplasms' OR 'mammary tumor' OR 'mammary tumor cell' OR 'mammary tumorigenesis' OR 'mammary tumour' OR 'mammary tumour cell' OR 'mass in the breast' OR 'masses in the breast' OR 'neoplasia of the breast' OR 'neoplasm of the breast' OR 'neoplasm of the mammary gland' OR 'neoplastic breast' OR 'neoplastic mammary' OR 'neoplastic mammary gland' OR 'tumor of the breast' OR 'tumor of the female breast' OR 'tumor of the male breast' OR 'tumor of the mammary gland' OR 'tumorigenesis of the breast' OR 'tumorigenesis of the mammary gland' OR 'tumour of the male breast' OR 'unilateral breast neoplasm' OR 'unilateral breast neoplasms' OR 'unilateral breast tumor' OR 'lung tumor'/exp OR 'broncho-pulmonary neoplasm' OR 'broncho-pulmonary tumor' OR 'bronchopulmonary neoplasia' OR 'bronchopulmonary neoplasm' OR 'bronchopulmonary tumor' OR 'lung neoplasia' OR 'lung neoplasm' OR 'lung neoplasms' OR 'lung tumor' OR 'lung tumorigenesis' OR 'lung tumour' OR 'neoplasia of the lung' OR 'neoplastic lung' OR 'pulmonary neoplasia' OR 'pulmonary neoplasm' OR 'pulmonary tumor' OR 'pulmonary tumorigenesis' OR 'pulmonary tumour' OR 'tumor of the lung' OR 'tumor, lung' OR 'tumorigenesis in the lung' OR 'tumour, lung' OR 'colorectal tumor'/exp OR 'colorectal neoplasia' OR 'colorectal neoplasm' OR 'colorectal neoplasms' OR 'colorectal tumor' OR 'colorectal tumorigenesis' OR 'colorectal tumour' OR 'neoplastic colorectal' OR 'tumor, colorectal' OR 'tumour, colorectal') AND ('implementation science'/exp OR 'implementation research' OR 'implementation science' OR 'implementation scientific research') AND [2004-2024]/py | 795 |
| 2 | ('asia'/exp OR asia) AND [2004-2024]/py | 13,63,443 |
| 3 | #1 AND #2 | 147 (included) |
| **Search strategy 4- Scopus** | | |
| 1 | ( INDEXTERMS ( "implementation science" ) OR INDEXTERMS ( "health plan implementation" ) ) OR ( TITLE-ABS ( "implementation science*" ) OR TITLE-ABS ( "health plan implementation*" ) OR TITLE-ABS ( implementation ) OR TITLE-ABS ( implementing ) OR TITLE-ABS ( implemented ) OR TITLE-ABS ( operations ) OR TITLE-ABS ( delivery ) OR TITLE-ABS ( deliver ) OR TITLE-ABS ( "translational science" ) OR TITLE-ABS ( "translational research" ) OR TITLE-ABS ( "translational medical research" ) OR TITLE-ABS ( "quality improvement" ) OR TITLE-ABS ( "task shifting" ) OR TITLE-ABS ( policy ) OR TITLE-ABS ( monitoring ) OR TITLE-ABS ( evaluation ) OR TITLE-ABS ( "implementation process evaluation" ) OR TITLE-ABS ( "impact evaluation" ) OR TITLE-ABS ( "effectiveness, implementation" ) OR TITLE-ABS ( "hybrid research" ) OR TITLE-ABS ( "implementation studies" ) OR TITLE-ABS ( "applied research" ) OR TITLE-ABS ( "practice based research" ) OR TITLE-ABS ( "operational research" ) OR TITLE-ABS ( "applied implementation science" ) OR TITLE-ABS ( "outcome evaluation" ) OR TITLE-ABS ( "evidence based practice" ) OR TITLE-ABS ( "policy implementation" ) OR TITLE-ABS ( "stakeholder engagement" ) OR TITLE-ABS ( "knowledge translation" ) OR TITLE-ABS ( "contextual factors" ) OR TITLE-ABS ( "barriers and facilitators" ) OR TITLE-ABS ( "implementation strategies" ) OR TITLE-ABS ( "implementation outcomes" ) OR TITLE-ABS ( "real world implementation" ) OR TITLE-ABS ( "implementation framework" ) OR TITLE-ABS ( "implementation fidelity" ) OR TITLE-ABS ( "implementation interventions" ) OR TITLE-ABS ( "implementation research methods" ) ) | 13,024,611 |
| 2 | ( INDEXTERMS ( "breast neoplasms" ) OR INDEXTERMS ( "lung neoplasms" ) OR INDEXTERMS ( "colorectal neoplasms" ) ) OR ( TITLE-ABS ( "breast neoplasm*" ) OR TITLE-ABS ( "neoplasm*, breast" ) OR TITLE-ABS ( "breast tumors*" ) OR TITLE-ABS ( "breast cancer*" ) OR TITLE-ABS ( "cancer*, breast" ) OR TITLE-ABS ( "mammary cancer*" ) OR TITLE-ABS ( "cancer, mammary*" ) OR TITLE-ABS ( "malignant neoplasm of breast*" ) OR TITLE-ABS ( "breast malignant neoplasm*" ) OR TITLE-ABS ( "breast malignant tumor*" ) OR TITLE-ABS ( "cancer of the breast*" ) OR TITLE-ABS ( "human mammary carcinoma*" ) OR TITLE-ABS ( "breast carcinoma" ) OR TITLE-ABS ( "carcinoma*, breast" ) OR TITLE-ABS ( "mammary neoplasm" ) OR TITLE-ABS ( "breast malignancy" ) OR TITLE-ABS ( "pulmonary neoplasm*" ) OR TITLE-ABS ( "neoplasms, lung*" ) OR TITLE-ABS ( "lung neoplasm*" ) OR TITLE-ABS ( "neoplasm*, lung" ) OR TITLE-ABS ( "neoplasm, pulmonary*" ) OR TITLE-ABS ( "lung cancer*" ) OR TITLE-ABS ( "cancer, lung*" ) OR TITLE-ABS ( "pulmonary cancer*" ) OR TITLE-ABS ( "cancer, pulmonary*" ) OR TITLE-ABS ( "cancer of the lung*" ) OR TITLE-ABS ( "pulmonary carcinoma" ) OR TITLE-ABS ( "lung carcinoma" ) OR TITLE-ABS ( "bronchogenic carcinoma" ) OR TITLE-ABS ( "bronchial carcinoma" ) OR TITLE-ABS ( "bronchial cancer" ) OR TITLE-ABS ( "pulmonary malignancy" ) OR TITLE-ABS ( "lung malignancy" ) OR TITLE-ABS ( "pulmonary tumor" ) OR TITLE-ABS ( "bronchogenic tumor" ) OR TITLE-ABS ( "bronchial tumor" ) OR TITLE-ABS ( "respiratory cancer" ) OR TITLE-ABS ( "pulmonary adenocarcinoma" ) OR TITLE-ABS ( "small cell lung cancer" ) OR TITLE-ABS ( "non-small cell lung cancer" ) OR TITLE-ABS ( "adenocarcinoma of the lung" ) OR TITLE-ABS ( "squamous cell carcinoma of the lung" ) OR TITLE-ABS ( "bronchoalveolar carcinoma" ) OR TITLE-ABS ( mesothelioma ) OR TITLE-ABS ( "colorectal neoplasm*" ) OR TITLE-ABS ( "neoplasm*, colorectal*" ) OR TITLE-ABS ( "colorectal tumor*" ) OR TITLE-ABS ( "tumor, colorectal*" ) OR TITLE-ABS ( "colorectal cancer*" ) OR TITLE-ABS ( "cancer*, colorectal" ) OR TITLE-ABS ( "colorectal carcinoma*" ) OR TITLE-ABS ( "carcinoma*, colorectal*" ) OR TITLE-ABS ( "bowel cancer" ) OR TITLE-ABS ( "colon cancer" ) OR TITLE-ABS ( "rectal cancer" ) OR TITLE-ABS ( "intestinal cancer" ) OR TITLE-ABS ( "colonic carcinoma" ) OR TITLE-ABS ( "rectal carcinoma" ) OR TITLE-ABS ( "large bowel carcinoma" ) OR TITLE-ABS ( "colorectal adenocarcinoma" ) OR TITLE-ABS ( "intestinal carcinoma" ) OR TITLE-ABS ( "colon adenocarcinoma" ) OR TITLE-ABS ( "rectal adenocarcinoma" ) OR TITLE-ABS ( "colorectal neoplasm" ) OR TITLE-ABS ( "large intestine cancer" ) OR TITLE-ABS ( "lower gastrointestinal cancer" ) OR TITLE-ABS ( "gastrointestinal malignancy" ) OR TITLE-ABS ( "bowel malignancy" ) OR TITLE-ABS ( "intestinal malignancy" ) ) | 1,190,257 |
| 3 | INDEXTERMS ( asia ) OR ( TITLE-ABS ( asia ) OR TITLE-ABS ( "northern asia" ) OR TITLE-ABS ( "north asia" ) OR TITLE-ABS ( "far east" ) OR TITLE-ABS ( "eastern asia" ) OR TITLE-ABS ( "east asia" ) OR TITLE-ABS ( "western asia" ) OR TITLE-ABS ( "southeastern asia" ) OR TITLE-ABS ( "southeast asia" ) OR TITLE-ABS ( "asia, central" ) OR TITLE-ABS ( "central asia" ) OR TITLE-ABS ( "south* asia" ) OR TITLE-ABS ( "asia, south" ) OR TITLE-ABS ( "british indian ocean territory" ) OR TITLE-ABS ( bangladesh ) OR TITLE-ABS ( india ) OR TITLE-ABS ( bharat ) OR TITLE-ABS ( "republic of india" ) OR TITLE-ABS ( myanma* ) OR TITLE-ABS ( burma ) OR TITLE-ABS ( "khmer republic" ) OR TITLE-ABS ( cambodia ) OR TITLE-ABS ( kampuchea ) OR TITLE-ABS ( "people&apos;s republic of china" ) OR TITLE-ABS ( "mainland china" ) OR TITLE-ABS ( sinkiang ) OR TITLE-ABS ( "inner mongolia" ) OR TITLE-ABS ( "netherlands east indies" ) OR TITLE-ABS ( "east indies" ) OR TITLE-ABS ( "west irian" ) OR TITLE-ABS ( "new guinea, indonesian" ) OR TITLE-ABS ( "new guinea, west" ) OR TITLE-ABS ( "indonesian new guinea" ) OR TITLE-ABS ( "irian jaya" ) OR TITLE-ABS ( timor ) OR TITLE-ABS ( java ) OR TITLE-ABS ( bali ) OR TITLE-ABS ( sumatra ) OR TITLE-ABS ( celebes ) OR TITLE-ABS ( sulawesi ) OR TITLE-ABS ( "malay archipelago" ) OR TITLE-ABS ( madura ) OR TITLE-ABS ( "bonin islands" ) OR TITLE-ABS ( malaya ) OR TITLE-ABS ( "federation of malaya" ) OR TITLE-ABS ( sabah ) OR TITLE-ABS ( sarawak ) OR TITLE-ABS ( "malay peninsula" ) OR TITLE-ABS ( "federal democratic republic of nepal" ) OR TITLE-ABS ( "islamic republic of pakistan" ) OR TITLE-ABS ( philipines ) OR TITLE-ABS ( "kingdom of thailand" ) OR TITLE-ABS ( siam ) OR TITLE-ABS ( "viet nam" ) OR TITLE-ABS ( "vietnam, republic of" ) OR TITLE-ABS ( "north vietnam" ) ) | 1,179,020 |
| 4 | #1 AND #2 AND #3 | 854 (included) |
| **Search strategy 5 – Web of Science** | | |
| 1 | (ALL="Implementation Science" OR ALL="Health Plan Implementation") OR ((TI="Implementation Science*" OR AB="Implementation Science*") OR (TI="Health Plan Implementation*" OR AB="Health Plan Implementation*") OR (TI=Implementation OR AB=Implementation) OR (TI=implementing OR AB=implementing) OR (TI=implemented OR AB=implemented) OR (TI=operations OR AB=operations) OR (TI=delivery OR AB=delivery) OR (TI=deliver OR AB=deliver) OR (TI="translational science" OR AB="translational science") OR (TI="translational research" OR AB="translational research") OR (TI="Translational Medical Research" OR AB="Translational Medical Research") OR (TI="quality improvement" OR AB="quality improvement") OR (TI="task shifting" OR AB="task shifting") OR (TI=policy OR AB=policy) OR (TI=monitoring OR AB=monitoring) OR (TI=evaluation OR AB=evaluation) OR (TI="implementation process evaluation" OR AB="implementation process evaluation") OR (TI="impact evaluation" OR AB="impact evaluation") OR (TI="effectiveness, implementation" OR AB="effectiveness, implementation") OR (TI="hybrid research" OR AB="hybrid research") OR (TI="implementation studies" OR AB="implementation studies") OR (TI="applied research" OR AB="applied research") OR (TI="practice based research" OR AB="practice based research") OR (TI="operational research" OR AB="operational research") OR (TI="applied implementation science" OR AB="applied implementation science") OR (TI="outcome evaluation" OR AB="outcome evaluation") OR (TI="evidence based practice" OR AB="evidence based practice") OR (TI="policy implementation" OR AB="policy implementation") OR (TI="stakeholder engagement" OR AB="stakeholder engagement") OR (TI="knowledge translation" OR AB="knowledge translation") OR (TI="contextual factors" OR AB="contextual factors") OR (TI="barriers and facilitators" OR AB="barriers and facilitators") OR (TI="implementation strategies" OR AB="implementation strategies") OR (TI="implementation outcomes" OR AB="implementation outcomes") OR (TI="real world implementation" OR AB="real world implementation") OR (TI="implementation framework" OR AB="implementation framework") OR (TI="Implementation fidelity" OR AB="Implementation fidelity") OR (TI="Implementation interventions" OR AB="Implementation interventions") OR (TI="Implementation research methods" OR AB="Implementation research methods")) | 9,907,157 |
| 2 | (ALL="Breast Neoplasms" OR ALL="Lung Neoplasms" OR ALL="Colorectal Neoplasms") OR ((TI="Breast Neoplasm*" OR AB="Breast Neoplasm*") OR (TI="Neoplasm*, Breast" OR AB="Neoplasm*, Breast") OR (TI="Breast Tumors*" OR AB="Breast Tumors*") OR (TI="Breast Cancer*" OR AB="Breast Cancer*") OR (TI="Cancer*, Breast" OR AB="Cancer*, Breast") OR (TI="Mammary Cancer*" OR AB="Mammary Cancer*") OR (TI="Cancer, Mammary*" OR AB="Cancer, Mammary*") OR (TI="Malignant Neoplasm of Breast*" OR AB="Malignant Neoplasm of Breast*") OR (TI="Breast Malignant Neoplasm*" OR AB="Breast Malignant Neoplasm*") OR (TI="Breast Malignant Tumor*" OR AB="Breast Malignant Tumor*") OR (TI="Cancer of the Breast*" OR AB="Cancer of the Breast*") OR (TI="Human Mammary Carcinoma*" OR AB="Human Mammary Carcinoma*") OR (TI="Breast Carcinoma" OR AB="Breast Carcinoma") OR (TI="Carcinoma*, Breast" OR AB="Carcinoma*, Breast") OR (TI="mammary neoplasm" OR AB="mammary neoplasm") OR (TI="breast malignancy" OR AB="breast malignancy") OR (TI="Pulmonary Neoplasm*" OR AB="Pulmonary Neoplasm*") OR (TI="Neoplasms, Lung*" OR AB="Neoplasms, Lung*") OR (TI="Lung Neoplasm*" OR AB="Lung Neoplasm*") OR (TI="Neoplasm*, Lung" OR AB="Neoplasm*, Lung") OR (TI="Neoplasm, Pulmonary*" OR AB="Neoplasm, Pulmonary*") OR (TI="Lung Cancer*" OR AB="Lung Cancer*") OR (TI="Cancer, Lung*" OR AB="Cancer, Lung*") OR (TI="Pulmonary Cancer*" OR AB="Pulmonary Cancer*") OR (TI="Cancer, Pulmonary*" OR AB="Cancer, Pulmonary*") OR (TI="Cancer of the Lung*" OR AB="Cancer of the Lung*") OR (TI="pulmonary carcinoma" OR AB="pulmonary carcinoma") OR (TI="lung carcinoma" OR AB="lung carcinoma") OR (TI="bronchogenic carcinoma" OR AB="bronchogenic carcinoma") OR (TI="bronchial carcinoma" OR AB="bronchial carcinoma") OR (TI="bronchial cancer" OR AB="bronchial cancer") OR (TI="pulmonary malignancy" OR AB="pulmonary malignancy") OR (TI="lung malignancy" OR AB="lung malignancy") OR (TI="pulmonary tumor" OR AB="pulmonary tumor") OR (TI="bronchogenic tumor" OR AB="bronchogenic tumor") OR (TI="bronchial tumor" OR AB="bronchial tumor") OR (TI="respiratory cancer" OR AB="respiratory cancer") OR (TI="pulmonary adenocarcinoma" OR AB="pulmonary adenocarcinoma") OR (TI="small cell lung cancer" OR AB="small cell lung cancer") OR (TI="non-small cell lung cancer" OR AB="non-small cell lung cancer") OR (TI="adenocarcinoma of the lung" OR AB="adenocarcinoma of the lung") OR (TI="squamous cell carcinoma of the lung" OR AB="squamous cell carcinoma of the lung") OR (TI="bronchoalveolar carcinoma" OR AB="bronchoalveolar carcinoma") OR (TI=mesothelioma OR AB=mesothelioma) OR (TI="Colorectal Neoplasm*" OR AB="Colorectal Neoplasm*") OR (TI="Neoplasm*, Colorectal*" OR AB="Neoplasm*, Colorectal*") OR (TI="Colorectal Tumor*" OR AB="Colorectal Tumor*") OR (TI="Tumor, Colorectal*" OR AB="Tumor, Colorectal*") OR (TI="Colorectal Cancer*" OR AB="Colorectal Cancer*") OR (TI="Cancer*, Colorectal" OR AB="Cancer*, Colorectal") OR (TI="Colorectal Carcinoma*" OR AB="Colorectal Carcinoma*") OR (TI="Carcinoma*, Colorectal*" OR AB="Carcinoma*, Colorectal*") OR (TI="bowel cancer" OR AB="bowel cancer") OR (TI="colon cancer" OR AB="colon cancer") OR (TI="rectal cancer" OR AB="rectal cancer") OR (TI="intestinal cancer" OR AB="intestinal cancer") OR (TI="colonic carcinoma" OR AB="colonic carcinoma") OR (TI="rectal carcinoma" OR AB="rectal carcinoma") OR (TI="large bowel carcinoma" OR AB="large bowel carcinoma") OR (TI="colorectal adenocarcinoma" OR AB="colorectal adenocarcinoma") OR (TI="intestinal carcinoma" OR AB="intestinal carcinoma") OR (TI="colon adenocarcinoma" OR AB="colon adenocarcinoma") OR (TI="rectal adenocarcinoma" OR AB="rectal adenocarcinoma") OR (TI="colorectal neoplasm" OR AB="colorectal neoplasm") OR (TI="large intestine cancer" OR AB="large intestine cancer") OR (TI="lower gastrointestinal cancer" OR AB="lower gastrointestinal cancer") OR (TI="gastrointestinal malignancy" OR AB="gastrointestinal malignancy") OR (TI="bowel malignancy" OR AB="bowel malignancy") OR (TI="intestinal malignancy" OR AB="intestinal malignancy")) | 1,024,220 |
| 3 | ALL=Asia OR ((TI=Asia OR AB=Asia) OR (TI="Northern Asia" OR AB="Northern Asia") OR (TI="North Asia" OR AB="North Asia") OR (TI="Far East" OR AB="Far East") OR (TI="Eastern Asia" OR AB="Eastern Asia") OR (TI="East Asia" OR AB="East Asia") OR (TI="Western Asia" OR AB="Western Asia") OR (TI="Southeastern Asia" OR AB="Southeastern Asia") OR (TI="Southeast Asia" OR AB="Southeast Asia") OR (TI="Asia, Central" OR AB="Asia, Central") OR (TI="Central Asia" OR AB="Central Asia") OR (TI="South* Asia" OR AB="South* Asia") OR (TI="Asia, South" OR AB="Asia, South") OR (TI="British Indian Ocean Territory" OR AB="British Indian Ocean Territory") OR (TI=Bangladesh OR AB=Bangladesh) OR (TI=India OR AB=India) OR (TI=Bharat OR AB=Bharat) OR (TI="Republic of India" OR AB="Republic of India") OR (TI=Myanma* OR AB=Myanma*) OR (TI=Burma OR AB=Burma) OR (TI="Khmer Republic" OR AB="Khmer Republic") OR (TI=Cambodia OR AB=Cambodia) OR (TI=Kampuchea OR AB=Kampuchea) OR (TI="People's Republic of China" OR AB="People's Republic of China") OR (TI="Mainland China" OR AB="Mainland China") OR (TI=Sinkiang OR AB=Sinkiang) OR (TI="Inner Mongolia" OR AB="Inner Mongolia") OR (TI="Netherlands East Indies" OR AB="Netherlands East Indies") OR (TI="East Indies" OR AB="East Indies") OR (TI="West Irian" OR AB="West Irian") OR (TI="New Guinea, Indonesian" OR AB="New Guinea, Indonesian") OR (TI="New Guinea, West" OR AB="New Guinea, West") OR (TI="Indonesian New Guinea" OR AB="Indonesian New Guinea") OR (TI="Irian Jaya" OR AB="Irian Jaya") OR (TI=Timor OR AB=Timor) OR (TI=Java OR AB=Java) OR (TI=Bali OR AB=Bali) OR (TI=Sumatra OR AB=Sumatra) OR (TI=Celebes OR AB=Celebes) OR (TI=Sulawesi OR AB=Sulawesi) OR (TI="Malay Archipelago" OR AB="Malay Archipelago") OR (TI=Madura OR AB=Madura) OR (TI="Bonin Islands" OR AB="Bonin Islands") OR (TI=Malaya OR AB=Malaya) OR (TI="Federation of Malaya" OR AB="Federation of Malaya") OR (TI=Sabah OR AB=Sabah) OR (TI=Sarawak OR AB=Sarawak) OR (TI="Malay Peninsula" OR AB="Malay Peninsula") OR (TI="Federal Democratic Republic of Nepal" OR AB="Federal Democratic Republic of Nepal") OR (TI="Islamic Republic of Pakistan" OR AB="Islamic Republic of Pakistan") OR (TI=Philipines OR AB=Philipines) OR (TI="Kingdom of Thailand" OR AB="Kingdom of Thailand") OR (TI=Siam OR AB=Siam) OR (TI="Viet Nam" OR AB="Viet Nam") OR (TI="Vietnam, Republic of" OR AB="Vietnam, Republic of") OR (TI="North Vietnam" OR AB="North Vietnam")) | 1,105,895 |
| 4 | #1 AND #2 AND #3 | 1285 (included) |
| **Search strategy 6 – ScienceDirect** | | |
|  | Implementation research on common cancers (Lung, Breast, and Colorectal) in Asia | 617 (included) |
| **Search strategy 7 – ProQuest** [Thesis and Dissertation] | | |
|  | Implementation research on common cancers (Lung, Breast, and Colorectal) in Asia  Included | 4590  140 (included) |
| **Search strategy 8 – Shodhganga** [Thesis and Dissertation] | | |
|  | “Implementation” AND “Research” “Common cancers (Lung, Breast, and Colorectal)” AND “Asia”  Included | 18805  0 (included) |
| **Search strategy 9 – Google Scholar** | | |
|  | Implementation research on common cancers (Lung, Breast, and Colorectal) in Asia  Included | 17,300  31 (included) |
