# Supplementary material for: Implementation research on common cancers (lung, breast, and colorectal) in Asia – a systematic review
Source: Front Oncol. 2025 Nov 7;15:1671298. doi: 10.3389/fonc.2025.1671298 (PMC12634338; doi:10.3389/fonc.2025.1671298)
Supplement: Supplementary Table 2 — Risk of bias assessment. [file Table2.docx]

**Table S2:** StaRI checklist (Risk of bias assessment)

| **Studies** | **Implementation score** | **Intervention score** | **Risk of bias*** |
| --- | --- | --- | --- |
| Zhang et al. (2019) | 18 | 20 | Low |
| Yang et al. (2017) | 19 | 15 | Low |
| Xiao et al. (2023) | 21 | 18 | Low |
| Ghoshal et al. (2021) | 16 | 15 | Low |
| Ma et al. (2012) | 20 | 16 | Low |
| Yeoh et al. (2018) | 19 | 17 | Low |
| Schliemann et al. (2020) | 21 | 16 | Low |
| Schliemann et al. (2023) | 21 | 16 | Low |
| Luo et al. (2021) | 18 | 16 | Low |
| Gong et al. (2018) | 20 | 15 | Low |
| Meng W et al. (2009) | 15 | 15 | Low |

***Risk of bias grading (based on total score out of 27):**

- **Low**: ≥ 20 items
- **Moderate**: 15–19 items
- **High**: < 15 items

**#StaRI checklist:** https://www.equator-network.org/reporting-guidelines/stari-statement/
